# Knowledge of Neonatal Danger Signs and Its Associated Factors among Mothers Attending Child Vaccination Centers at Sheko District in Southwest Ethiopia

**DOI:** 10.1155/2020/4091932

**Published:** 2020-05-14

**Authors:** Tewodros Yosef, Tadesse Nigussie, Adane Asefa

**Affiliations:** Department of Public Health, College of Medicine and Health Sciences, Mizan-Tepi University, Mizan Teferi, Ethiopia

## Abstract

**Background:**

Even though a great improvement in the last twenty years, the problem of newborn deaths is still remaining. In 2017 alone, an estimated 2.5 million neonatal deaths occurred worldwide, around 39 percent of all deaths in sub-Saharan Africa. Early detection of neonatal illness is an important step towards improving newborn survival. If mothers know the appropriate manifestations of the causes of death in newborns (neonatal danger signs), it is possible to avert related mortality, because of the health-seeking behavior of mothers highly relies on their knowledge of neonatal danger signs.

**Objective:**

To assess knowledge of neonatal danger signs and its associated factors among mothers attending child vaccination centers at Sheko District in Southwest Ethiopia.

**Methods:**

A facility-based cross-sectional study was conducted among 351 mothers who attended health centers for child vaccination in Sheko District from March 17 to April 30, 2018. A consecutive sampling method was used to select study participants. Data were collected by using structured questionnaires through face-to-face interviews. Data were entered using EPI-DATA version 3.1 and analysed using SPSS version 21.

**Results:**

Of the 351 mothers interviewed, 39% (137) had good knowledge of neonatal danger signs. The study also found that mothers aged 29-40 years (AOR = 2.37, 95% CI [1.35-4.17], *P* = 0.003), educational status of primary and above (AOR = 2.68, 95% CI [1.48-4.88], *P* = 0.001), attending ≥ 4 antenatal care visits during pregnancy (AOR = 3.57, 95% CI [2.10-6.06], *P* < 0.001), and history of postnatal attendance after birth (AOR = 2.33, 95% CI [1.16-4.65], *P* = 0.017) were significantly associated with good knowledge of neonatal danger signs.

**Conclusion:**

The proportion of mothers with good knowledge of neonatal danger signs was remarkably low. Since the problem is a public health importance in developing countries, particularly in Ethiopia, which determines future generations. Great efforts are needed to create awareness for mothers on the importance of the early identifying neonatal danger signs plus to avert the high magnitude of neonatal mortality.

## 1. Introduction

Despite progress over the past two decades, neonatal mortality remains a problem. Globally, an estimated 2.5 million newborn deaths occurred in 2017 alone, mostly from preventable causes. Sub-Saharan Africa accounted for 39 per cent of all deaths [1]. The majority of newborn deaths occur in developing countries, where most newborn deaths occur at home [2–4]. Reducing neonatal mortality is among the health-related indicators of sustainable development goal (SDG) to be achieved by 2030 [5]. The global neonatal mortality rate declined from 31 deaths per 1,000 live births in 2000 to 18 deaths in 2017. But still there are disparities in reducing neonatal mortality across regions and countries. One hundred eighteen countries were below the target achievement in 2017. More efforts are needed to accelerate in about 50 countries, mainly in sub-Saharan Africa, including Ethiopia, to meet the target by 2030 [6].

Neonatal mortality contributes to nearly half of the under-five mortality in Ethiopia [7]. Neonatal mortality declined from 49 deaths per 1,000 live births in 2000 to 29 deaths per 1,000 births in 2016, a reduction of 41% over the past 16 years [8]. However, the patterns of neonatal mortality have persisted high in Ethiopia in spite of many efforts being applied to decrease this adverse trend [9].

Early detection of neonatal illness is an important step towards improving newborn survival [9, 10]. A mother is the nearest person to a neonate to identify, present, and manage the neonates' problem, which ensures that neonates can lead a healthy life [11]. If mothers know appropriate manifestations of the causes of death in newborns (neonatal danger signs), it is possible to avert related neonatal mortality [12]. Because of the health-seeking behavior of mothers highly relies on their knowledge of neonatal danger signs [13].

Different studies conducted previously regarding mothers' level of knowledge of neonatal danger signs and reported that 11.67% in Woldiya [9], 18.2% in Gondar [13], 20.3% in Ambo [14], 32.9% in Harar [15], 40.9% in Arba Minch [16], 50.3% in Haramaya [17] from studies conducted in Ethiopia, 15.5% in Kenya [18], 28.1% in Ghana [19], and 48.18% in Nepal [20] of mothers had good knowledge of neonatal danger signs.

The factors that influence mothers' level of knowledge about neonatal danger signs are varied and may include age, maternal and husband education, occupational status, place of residence, history and frequency of antenatal care (ANC) visits, history of postnatal care (PNC) visits, parity, and place of delivery [9, 10, 14–16, 18, 20–24].

Neonatal danger signs has become a substantial problem in many developing countries like Ethiopia [13]. A significant tackling of the problem needs good knowledge of neonatal danger signs among mothers. Despite studies done in Ethiopia regarding this issue [9, 13, 15, 17, 23], almost all studies were done in more urban societies. However, this study was done in more rural and semi-urban communities with extreme socioeconomic and cultural differences compared to previous studies done in Ethiopia. Therefore, this study aimed to assess knowledge of neonatal danger signs and its associated factors among mothers attending child vaccination centers at Sheko District in Southwest Ethiopia.

## 2. Methods

### 2.1. Study Design, Period, and Setting

The facility-based cross-sectional study was conducted at Sheko District health facilities in Bench-Sheko Zone, Southwest Ethiopia from March 17 to April 30, 2018. Sheko District is located at 580 km southwest of Addis Ababa, the capital city of Ethiopia. The district has 4 health centers, which provide health services for a catchment population of approximately 34,237.

### 2.2. Populations

The source of the populations was all mothers who gave birth in the last one year and attending vaccination during the study period. The study population was selected mothers who gave birth in the last one year and attending vaccination during the study period.

### 2.3. Sample Size and Sampling Technique

The sample size was determined using a single population proportion formula (*n* = ((*z*_*α*/2_)^2^*p*(1 − *p*))/*d*^2^) [25], based on the assumptions of 95% confidence level, 5% margin of error, and proportion of good knowledge of neonatal danger signs (31.32%), which was taken from a previous study done at Wolkitie District, Ethiopia [21], and adding 10% nonresponse rate, the final computed sample size was 363. Based on the number of mothers appointed for immunization at each health center, the total sample was proportionally allocated. Finally, study participants were consecutively enrolled in study based on their arrival at the health centers for immunization. Mothers were interviewed on their entry before getting services to minimize bias.

### 2.4. Data Collection Tool and Procedures

A structured questionnaire was used to collect the data through face-to-face interviews. The questionnaire was adapted from a previous study [21]. The questionnaire was translated to Amharic and back to English to assure its consistency. It was also pretested on five percent of the sample, and modifications such as readability, grammar, and order of question were made. Training was given to data collectors and supervisors concerning the objective and process of data collection and to discuss the presence of an ambiguous question in the questionnaire.

### 2.5. Study Variables

The dependent variable was knowledge of neonatal danger signs. The independent variables were sociodemographic variables (age, occupation, marital status, education level, and place of residence), and reproductive health variables (parity, place of delivery, antenatal care, and postnatal care services).

### 2.6. Operational Definitions

- Neonatal danger signs are signs that sick neonates show as stated by World Health Organization (WHO), which include not able to feed, or stopped feeding well, convulsed or fitted since birth, fast breathing (two counts of 60 breaths or more in one minute), chest in drawing, high temperature (37.5°C or more), very low temperature (35.4°C or less), yellow soles, movement only when stimulated, or no movement even on stimulation and signs of local infection such as umbilicus redness or draining of pus, skin boils, or eyes draining pus [26].
- Knowledge of neonatal danger signs: mothers who mentioned at least three WHO neonatal danger signs were considered as having good knowledge, and those who mentioned less than three were considered as having poor knowledge [27].

### 2.7. Data Processing and Analysis

Data were entered into Epi-data version 3.1 and analysed using SPSS version 21. Descriptive statistics was done for important variables using tables and numerical summery measures such as mean and standard deviation (SD). A binary logistic regression analysis was done to determine the likelihood of an association between dependent and independent variables. Independent variables with a *P* value of <0.25 in the bivariate logistic regression model were included in the multivariable logistic regression. Finally, variables with a *P* value of <0.05 in the multivariable logistic regression were taken as factors associated with knowledge of neonatal danger signs. Multicollinearity between independent variables was checked, and the variance inflation factor was found acceptable (<2). The Hosmer-Lemeshow goodness-of-fit test indicated (*P* = 0.864) that the model was good enough to fit the data well.

## 3. Results

### 3.1. Sociodemographic Characteristics of Mothers

Of the 363 sample size recruited, 351 gave completed response to the interview, yielding a response rate of 96.7%. The mean age of mothers was 26.7(±5.05 SD) years, ranging from 18 to 40 years. Two hundred sixteen (61.5%) mothers had achieve primary school and above level of education (Table 1).

### 3.2. Reproductive History of Mothers

Three hundred-twenty (91.2%) mothers attended ANC follow-up for their last pregnancy. Seventy-one (22.2%) had the recommended ANC frequency by World Health Organization (≥4 visits during pregnancy). Two hundred eighty-six (81.5%) had postnatal care attendance during their last childbirth (Table 2).

### 3.3. Mothers' Knowledge of Neonatal Danger Signs (NDS)

Of the 351 respondents, 137 (39%) had good knowledge of neonatal danger signs. More than two-thirds (71.5%) and nearly one-tenths (11.7%) of mothers mentioned fever and yellowish discoloration of skin as neonatal danger signs, respectively (Table 3).

### 3.4. Factors Associated with Mothers' Knowledge of Neonatal Danger Signs

In the bivariate logistic regression analysis, age, educational status of mothers, place of residence, occupation, frequency of ANC, and history of PNC were associated with the outcome variable at *P* value of <0.25 and were included in the multivariable logistic regression analysis. In the multivariable logistic regression, age, educational status of mothers, frequency of ANC visits, and history of PNC attendance were significantly associated with mothers' knowledge of neonatal danger signs (Table 4).

## 4. Discussion

Early detection of neonatal illness is an important step towards improving newborn survival [9, 10]. If mothers know appropriate manifestations of neonatal danger signs, it is possible to avert related neonatal mortality [12], because the health-seeking behavior of mothers highly relies on their knowledge of neonatal danger signs [13]. Based on the above facts, we aimed to assess the knowledge of neonatal danger signs and its associated factors among mothers attending child vaccination centers at Sheko District in Southwest Ethiopia. As a result, the proportion of mothers with good knowledge of neonatal danger signs was found to be 39%, 95% CI (33.9%-44.1%). This finding was consistent with 40.9% in Arba Minch, Ethiopia [16].

This finding was higher than 11.67% in Woldiya [9], 18.2% in Gondar [13], 20.3% in Ambo [14], 32.9% in Harar [15] studies conducted in Ethiopia, 15.5% in Kenya [18], and 28.1% in Ghana [19]. However, it was lower than 48.18% in Nepal [20] and 50.3% in Haramaya, Ethiopia [17], of mothers who had good knowledge of neonatal danger signs. The variation observed compared to other studies could be due to the differences in sample size, operational definition used (since some studies use three and others use four, etc., criteria to say knowledgeable about neonatal danger signs) and methodology in general. In addition, socioeconomic, cultural, and educational profiles of the study population may create a significant variation.

Mothers aged 29-40 years have 2.4 times increased odds of having good knowledge of neonatal danger signs than mothers with age of 18-28 years. Being older was associated with good knowledge. This finding was supported by studies done in Woldiya, Ethiopia, and Nepal [9, 20]. This could be due to the more experiences from multiple parity and child rearing.

It is obvious that being educated is significantly associated with better awareness and health-seeking behavior of any disease conditions. Those mothers with primary and above levels of education have 2.7 times higher odds of having good knowledge of neonatal danger signs than those with no formal education. This finding was supported by previous studies [9, 13–16, 18, 22, 23].

In this study, having the recommended above ANC visits was strongly associated with good knowledge of neonatal danger signs. Mothers who had four or more ANC visits during pregnancy have 3.6 increased odds of having good knowledge of neonatal danger signs than those with fewer than four ANC visits, since mothers who have frequent ANC visits during pregnancy are more likely to have postnatal care. It could be due to the awareness creation conducted regarding neonatal danger signs during their PNC visits. This finding was consistent with studies done in Woldiya, Ethiopia, and Iraq [9, 28]. However a study done in Uganda revealed no association between women attending the recommended number of antenatal care visits and their knowledge of danger signs [10].

Mothers who had a history of postnatal care attendance after birth have 2.3 times higher odds of having good knowledge of neonatal danger signs. Having postnatal care attendances after childbirth was associated with good knowledge of neonatal danger signs. This finding was supported by studies done in Ambo and Arba Minch, Ethiopia [14, 16].

## 5. Conclusion

The proportion of mothers with good knowledge of neonatal danger signs was remarkably low. Since the problem is a public health importance in developing countries, particularly in Ethiopia, which determines future generations, great efforts are needed to create awareness for mothers on the importance of the early identification of neonatal danger signs to avert the high magnitude of neonatal mortality.

## Figures and Tables

**Table 1:** Sociodemographic characteristics of mothers at Sheko District health centers in Southwest Ethiopia.

Variables	Categories	Frequency	Percent
Age group	18-28	228	65
	29-40	123	35

Marital status	In marriage	303	86.3
	Out of marriage	48	13.7

Residence	Rural	144	41
	Urban	207	59

Mothers' education	No formal education	135	38.5
	Primary school and above	216	61.5

Occupation of mothers	Housewife	238	67.8
	Government worker	113	32.2

Husbands' education	No formal education	154	43.9
	Primary school and above	197	56.1

**Table 2:** Reproductive history of mothers at Sheko District health centers in Southwest Ethiopia.

Variables	Categories	Frequency	Percent
Parity (*n* = 351)	<2	156	44.4
	2-4	113	32.2
	>5	82	23.4

History of antenatal care attendance (*n* = 351)	Yes	320	91.2
	No	31	8.8

Frequency of antenatal care visit (*n* = 320)	1 visit	86	26.9
	2 visits	106	33.1
	3 visits	57	17.8
	≥4 visits	71	22.2

History of postnatal care attendance (*n* = 351)	Yes	286	81.5
	No	65	18.5

**Table 3:** Mothers' awareness of specific neonatal danger signs at Sheko District health centers, Southwest Ethiopia.

Neonatal danger signs	Categories	Frequency	Percent
Persistent vomiting	Yes	156	44.4
	No	195	55.6

Fever (high temperature)	Yes	251	71.5
	No	100	28.5

Difficulty of breathing	Yes	113	32.2
	No	238	67.8

Hypothermia (low temperature)	Yes	79	22.5
	No	272	77.5

Poor feeding	Yes	84	23.9
	No	267	76.1

Convulsion	Yes	72	20.5
	No	279	79.5

Lethargic	Yes	66	18.8
	No	285	81.2

Yellowish discoloration of skin	Yes	41	11.7
	No	310	88.3

**Table 4:** Factors associated with mothers' good knowledge of neonatal danger signs at Sheko District health centers in Southwest Ethiopia.

Variables	Categories	Knowledge of NDS		COR (95% CI)	AOR (95% CI)	*P* value
		Poor	Good			
Age group	18-28 years	145	83	1	1	
	29-40 years	69	54	1.37 (0.88-2.14)∗	2.37 (1.35-4.17)	**0.003**

Mothers' education	No formal education	101	34	1	1	
	Primary and above	113	103	2.71 (1.69-4.34)∗∗	2.68 (1.48-4.88)	**0.001**

Occupation	Housewife	160	78	1		
	Government worker	54	59	2.24 (1.42-3.54)∗∗	1.30 (0.76-2.22)	0.338

Residence	Rural	96	48	1	1	
	Urban	118	89	1.51 (0.97-2.35)∗	0.83 (0.48-1.44)	0.505

Antenatal care frequency	<4 visits	143	51	1	1	
	≥4 visits	52	76	4.10 (2.55-6.60)∗∗	3.57 (2.10-6.06)	**<0.001**

History of postnatal care attendance	Yes	182	104	1.81 (1.05-3.11)∗∗	2.33 (1.16-4.65)	**0.017**
	No	32	33	1	1	

AOR: adjusted odds ratio; CI: confidence interval; COR: crude odds ratio; NDS: neonatal danger signs. ^∗^Significant at a *P* value of <0.25, ^∗∗^significant at a *P* value of <0.05.

## Data Availability

The data set is handled by the corresponding author and can be provided upon request.

## Abbreviations

ANC: Antenatal care
AOR: Adjusted odds ratio
CI: Confidence interval
COR: Crude odds ratio
PNC: Postnatal care
SPSS: Statistical Package for Social Sciences
SD: Standard deviation.
